# Information processing speed modulation by electrical brain stimulation in multiple sclerosis: towards individually tailored protocols

**DOI:** 10.1093/braincomms/fcaf223

**Published:** 2025-06-06

**Authors:** Steffen Riemann, Michel Mittelstädt, Maurice Glatzki, Carlotta Zilges, Clara Wolff, Filip Niemann, Mandy Roheger, Agnes Flöel, Matthias Grothe, Marcus Meinzer

**Affiliations:** Department of Neurology, University Medicine Greifswald, Greifswald 17475, Germany; Department of Neurology, University Medicine Greifswald, Greifswald 17475, Germany; Department of Neurology, University Medicine Greifswald, Greifswald 17475, Germany; Department of Neurology, University Medicine Greifswald, Greifswald 17475, Germany; Department of Neurology, University Medicine Greifswald, Greifswald 17475, Germany; Department of Neurology, University Medicine Greifswald, Greifswald 17475, Germany; Department of Neurology, University Medicine Greifswald, Greifswald 17475, Germany; Department of Psychology, Ambulatory Assessment in Psychology, Carl Von Ossietzky University, Oldenburg 26129, Germany; Department of Neurology, University Medicine Greifswald, Greifswald 17475, Germany; Department of Neurology, University Medicine Greifswald, Greifswald 17475, Germany; Department of Neurology, University Medicine Greifswald, Greifswald 17475, Germany

**Keywords:** relapsing multiple sclerosis, information processing speed, focalized transcranial direct current stimulation, superior parietal cortex, Bayesian modelling

## Abstract

Information processing speed is a core cognitive process, highly relevant in everyday life and the most frequent and disabling cognitive symptom in patients with relapsing multiple sclerosis. Correlational evidence from brain imaging suggests involvement of the superior parietal lobe in the speed component of information processing, thereby providing a neurobiological foundation for neuromodulatory interventions. By using regionally specific, focalized transcranial direct current stimulation (tDCS) in healthy individuals and patients with relapsing multiple sclerosis, we provide causal evidence for superior parietal lobe involvement in information processing speed and identified a clinically relevant predictor of tDCS response in patients with relapsing multiple sclerosis. The study employed a registered, randomized, sham tDCS-controlled, three-way-blinded, cross-over trial and a mixed-factors design with eight arms [between-subjects: group (patients/healthy controls; *N* = 32/group); tDCS polarity (excitatory/inhibitory); within-subjects: stimulation (active/sham tDCS)]. Concurrently with tDCS (1.5 mA; active: 20 min; sham: 40 s), participants completed a computerized version of the Symbol Digit Modalities Test, the current gold standard for quantifying information processing speed impairment in patients with relapsing multiple sclerosis. Data were analysed in a Bayesian framework with generalized linear mixed models. Bayesian modelling provided strong causal evidence of bilateral superior parietal lobe involvement in information processing speed and a double dissociation of stimulation response in patients and controls (i.e. a significant three-way interaction of group × stimulation × polarity). Healthy individuals showed the expected canonical pattern of significantly reduced and increased response latency during anodal or cathodal tDCS, respectively. Across the patient groups, a reversed pattern was found and tDCS response was predicted by baseline Symbol Digit Modalities Test performance. More impaired patients benefited from cathodal tDCS, while less impaired patients benefited from anodal tDCS. For standardized Symbol Digit Modalities Test scores, the transition from beneficial to non-beneficial effects (anodal: < −0.58; cathodal: > −0.70) was consistent across the patient groups. tDCS was well tolerated, with no evidence for differences in mild adverse effects across groups and tDCS conditions. Blinding integrity was confirmed and behavioural outcomes were not explained by factors unrelated to tDCS. Our results provide direct causal evidence for superior parietal lobe involvement in information processing speed in health and disease and suggest that the degree of information processing speed impairment in the patients reflects compensatory or dysfunctional neuroplastic processes that can be counteracted by tDCS in a polarity-specific way. Identified standardized transition scores for the effectiveness of excitatory or inhibitory tDCS will inform future individually tailored stimulation protocols in patients with relapsing multiple sclerosis (trial registration: NCT04667221).

## Teaser

In a proof-of-concept study, Riemann *et al*. investigated if bilateral focal electrical brain stimulation of the superior parietal lobe can improve impaired information processing speed in people with relapsing multiple sclerosis. Results suggest that the initial degree of processing speed impairment determined the effectiveness of excitatory or inhibitory brain stimulation.

## Graphical Abstract

**Graphical Abstract:**
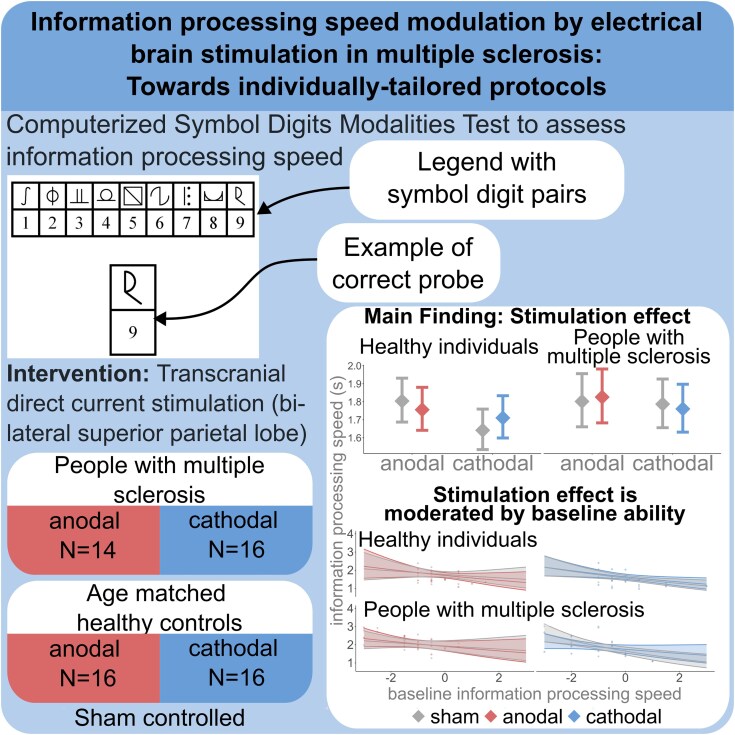

## Introduction

Multiple sclerosis (MS) is a chronic inflammatory disease resulting in progressive central nervous system demyelination and neurodegeneration.^1^ The relapsing form of MS (RMS) is the leading cause of non-traumatic neurologic disability in early adulthood worldwide and manifests as recurrent episodes of inflammation, followed by variable degrees of remission and progressive neurological dysfunction.^2^ Patients may suffer from physical and psychological symptoms, and up to 65% of patients present with cognitive impairment.^3^ Cognitive impairment predicts later disability, is one of the main reasons for reduced work productivity^4^ and is associated with reduced quality of life.^4-8^

The most prevalent and disabling cognitive symptom in people with RMS (pwRMS) is reduced information processing speed (IPS).^3,9^ IPS is a key cognitive process that comprises the ability to identify, discriminate, integrate, and make decisions about incoming information, and to swiftly respond at the behavioural level. According to the tri-factor model of IPS, sensory, cognitive and motor speed components can be distinguished that are subserved by a brain network comprised of sensory, frontoparietal and cerebellar regions.^10^ It is estimated that 27–51% of pwRMS patients suffer from IPS impairment, which has been linked to disease progression, but can also be the only symptom of a relapse.^3^

Drug treatment of the inflammatory neuropathology cannot directly target impaired IPS, and there are currently no approved medications for treating cognitive symptoms in pwRMS.^3,11^ This highlights the clinical relevance of exploring novel and evidence-based treatments that directly modulate activity and plasticity in the neural networks subserving IPS in pwRMS.^9^ A promising approach to achieve this is non-invasive transcranial direct current stimulation (tDCS), that administers weak electrical currents via scalp attached electrodes to the brain, to modulate neural excitability and plasticity in the human brain.^12^ tDCS has an excellent safety profile and has successfully been used to improve cognitive functions in healthy individuals and patients with neuropsychiatric disorders.^13-19^

To date, the majority of tDCS studies in pwRMS have targeted motor symptoms or fatigue and only two studies specifically investigated tDCS effects on IPS.^20,21^ These studies administered multisession tDCS to the prefrontal cortex, either as standalone treatment or in combination with cognitive training.^22,23^ Only the latter reported improved IPS immediately after the treatment relative to a control group, but no between-group effects 6 months later. These mixed effects of tDCS mirror those reported for other symptoms in pwRMS.^20,21^

Moreover, animal and human studies have demonstrated that behavioural and neural effects of multisession tDCS can be maintained for weeks or even months after the end of the intervention period.^24-27^ In this context, it is worth noting that reorganization of the functional brain networks supporting cognition in pwRMS is poorly understood, and upregulation of frontoparietal activity in pwRMS may reflect compensatory or dysfunctional neuroplasticity, depending on disease progression.^28-33^ This makes it difficult to predict the neural effects of specific tDCS montages, and unintended stimulation effects in multisession contexts bear the risk for inducing maladaptive neuroplasticity. Hence, there is currently an urgent need for conducting carefully designed proof-of-concept tDCS studies that investigate the potential of specific tDCS interventions to enhance IPS and to identify predictors of stimulation response, prior to implementation in time- and cost-intensive clinical trials, while minimizing the risk for the patients.

This was accomplished in the present study, by implementing a balanced and randomized cross-over trial that involved two experimental sessions, either with active (anodal or cathodal tDCS) or placebo (sham) tDCS. A single active tDCS session was chosen, because effects are completely reversible, thereby minimizing the risk for potential negative long-term effects.^13^ During each session, pwRMS and age- and sex-matched healthy individuals completed a computerized version of the Symbol Digit Modalities Test (SDMT), which is the current gold standard to determine the degree of processing speed impairment in pwRMS.^34^ tDCS was administered bilaterally to the superior parietal lobe (SPL), based on previous functional and diffusion imaging studies, suggesting specific involvement of this region in the speed component of IPS.^35,36^ A focalized tDCS setup was used that constrains the current delivery to the target regions and has been shown to induce regional- and task-specific neural modulation.^37-41^ By investigating polarity-specific effects of focal tDCS, we aimed to establish causal involvement of the SPL in IPS. Inclusion of a matched healthy control group served to determine the degree of IPS impairment in pwRMS (during sham tDCS) and to investigate the question if the hypothesized canonical anodal-excitatory, cathodal-inhibitory (AeCi) response to active tDCS in healthy control groups can be replicated in the patient groups. Based on functional imaging studies suggesting that frontoparietal regions may reflect compensatory or dysfunctional processes depending on disease progression, we also explored if tDCS response in the patients can be predicted by the degree of IPS impairment.^28-33^

## Materials and methods

This prospective, randomized, three-way-blinded (i.e. participants, staff conducting the experiment and evaluating outcomes), sham tDCS-controlled, cross-over trial was conducted in a mixed-factors design with eight arms, representing the between-subjects factors group (pwRMS, healthy controls) and tDCS polarity (anodal, cathodal), and the within-subjects factor stimulation (active, sham tDCS). The study was approved by the Medical Ethics Committee of the University Medicine Greifswald, conducted in accordance with the Helsinki Declaration, and the patient arm of the study was registered with ClinicalTrials.gov (NCT04667221).

### Participants

Thirty-two pwRMS (male/female: 11/21; mean (*M*) ± standard deviation (SD) age: 46.9 ± 11.3 years) were recruited via the out-patient clinic at the University Medicine Greifswald and self-help groups. Inclusion criteria comprised age ≥18 years, a specialist-confirmed diagnosis of RMS based on the revised McDonald criteria,^42^ no acute relapse and/or application of corticosteroids within the last 3 months prior to the experimental intervention, normal or corrected to normal vision, sufficient hand motor function to respond by button press on a computer keyboard and being a native German speaker. Exclusion criteria included other major medical or neuropsychiatric diseases (e.g. major depression) and contraindications for tDCS (e.g. metal implants, prior medical procedures involving head or spinal cord, head trauma with unconsciousness, history of epilepsy, convulsions, seizures, migraine and current pregnancy).^13^ The sample size was based on previous cross-over tDCS trials from our group in different neurological populations (note that these trials only involved comparison of anodal and sham tDCS, and larger effects were expected for the between group comparison of anodal and cathodal tDCS).^43,44^

Thirty-two healthy individuals were recruited from the local community and retrospectively matched to individual patients on a 1:1 basis by age and sex (male/female: 11/21; *M* ± SD age: 46.3 ± 11.0 years). This group was included to determine the degree of IPS impairment in the patients (during sham tDCS) and to investigate potential differences in stimulation response in the patients relative to healthy controls.

All participants provided written informed consent prior to study inclusion, completed a demographic questionnaire and a comprehensive neuropsychological baseline examination and were screened for depression and anxiety. Characteristics of patients and healthy controls, results of neuropsychological baseline assessments and additional questionnaires are detailed in Table 1.

**Table 1:** Demographic and clinical characteristics of patients and healthy controls, and details of the neuropsychological assessment^a^

	Control				pwRMS				Group differences			
	Anodal		Cathodal		Anodal		Cathodal		Anodal		Cathodal	
Sex (male/female)	4/12		7/9		4/10		6/10					
	*M*	SD	*M*	SD	*M*	SD	*M*	SD	SMD	95% CI	SMD	95% CI
Age	47.56	10.86	45.06	11.99	50.36	8.67	42.31	11.35	−0.28	[−3.83 to 3.26]	0.24	[−3.81 to 4.28]
Education (years)	18.34	2.93	17.12	2.91	13.43	1.84	16.25	3.32	1.98^b^	[1.09 to 2.87]	0.28	[−0.80 to 1.36]
Disease duration (years)					12.3	9.62	9.19	8.16				
EDSS					1.80	1.30	3.05	1.51				
SDMT total	52.75	7.02	55.62	10.46	38.21	9.39	44.75	12.51	1.77	[−1.16 to 4.71]	0.94	[−3.05 to 4.94]
VLMT learning	62.25	7.43	60.00	9.49	46.50	14.22	54.88	11.38	1.42	[−2.56 to 5.39]	0.49	[−3.14 to 4.12]
VLMT delayed recall	12.19	2.88	13.00	1.90	9.00	3.26	11.31	3.07	1.04^c^	[−0.05 to 2.14]	0.66	[−0.22 to 1.55]
VLMT forgetting	1.56	2.03	0.94	1.34	2.14	2.14	1.56	1.59	−0.28	[−1.02 to 0.47]	−0.42	[−0.93 to 0.09]
VLMT interference	0.12	0.34	0.50	1.15	6.36	2.06	6.25	1.91	−4.38^d^	[−4.89 to −3.87]	−3.65^d^	[−4.19 to −3.10]
EHI laterality quotient	72.83	43.15	84.33	15.95	59.40	32.35	66.63	23.86	0.35	[−13.43 to 14.13]	0.87	[−6.16 to 7.90]
TMT-A time	25.49	6.49	23.54	7.94	42.00	15.70	32.58	9.67	−1.41	[−5.60 to 2.78]	−1.02	[−4.09 to 2.04]
TMT-A errors	0.19	0.40	0.25	0.58	0.50	0.65	0.38	0.62	−0.58	[−0.77 to −0.39]	−0.22	[−0.42 to −0.01]
TMT-B time	59.76	15.03	51.76	18.55	86.94	34.56	76.61	35.61	−1.05	[−10.35 to 8.26]	−0.88	[−10.71 to 8.96]
TMT-B errors	0.19	0.40	0.19	0.40	0.50	0.85	0.31	0.48	−0.48	[−0.71 to −0.25]	−0.27	[−0.42 to −0.12]
FSMC cognition	19.94	4.80	20.06	7.62	34.71	6.01	36.50	6.57	−2.74	[−4.67 to −0.81]	−2.31	[−4.78 to 0.15]
FSMC motor	18.69	4.09	19.06	7.35	33.71	8.42	35.44	6.80	−2.32	[−4.64 to −0.01]	−2.31	[−4.77 to 0.14]
FSMC total	38.62	8.23	39.06	13.88	68.43	13.72	71.94	12.28	−2.68	[−6.66 to 1.30]	−2.51	[−7.05 to 2.03]
HADS-D depression	1.81	1.38	1.62	2.00	6.43	4.62	5.69	2.77	−1.40^b^	[−2.58 to −0.21]	−1.68	[−2.52 to −0.85]
HADS-D anxiety	4.38	2.45	3.88	2.50	6.29	2.92	7.25	3.59	−0.71^b^	[−1.67 to 0.25]	−1.09^b^	[−2.16 to −0.02]

*M*, mean. SD, standard deviation. SMD, standardized mean difference; 95%CI, 95% confidence interval; pwRMS, people with relapsing multiple sclerosis; EDSS, Expanded Disability Status Scale; VLMT, Verbal Learning and Memory Test; EHI, Edinburgh Handedness Inventory; TMT, Trail Making Test. FSMC, Fatigue Scale for Motor and Cognitive Functions; SDMT, Symbol Digit Modalities Test; HADS-D, Hospital Anxiety and Depression Scale—German version.

^a^Raw scores are reported; all healthy participants scored within age-corrected norms. Group differences (patients, controls) in the two stimulation arms are provided using SMD.

^b^Evidence ratio > 99.

^c^Evidence ratio > 19.

^d^Evidence ratio > 999.

**Figure 1:**
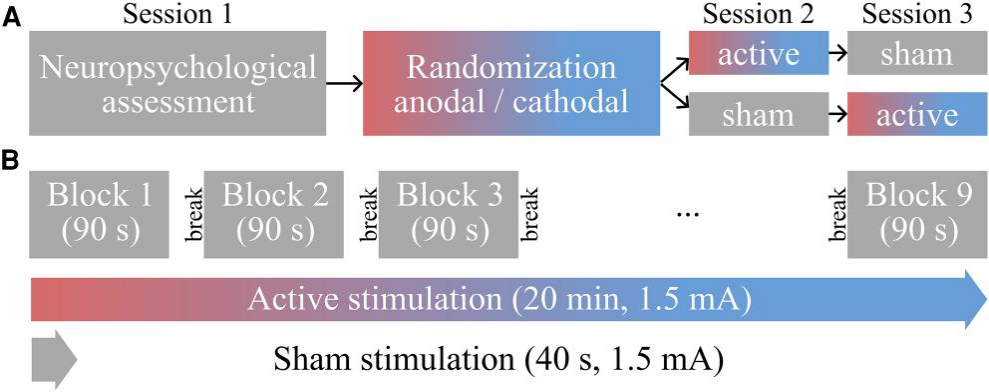
**Study overview.** (**A**) The study comprised three sessions: a neuropsychological baseline assessment, followed by two experimental sessions (either active or sham tDCS); stimulation order was counterbalanced in each participant group (i.e. 50% of participants received active tDCS first, followed by sham tDCS; 50% received sham first, followed by active stimulation). Stimulation sessions were separated by at least 1 week. (**B**) Procedure of the experimental sessions: nine blocks of the modified SDMT were completed with either active (anodal, cathodal) or sham stimulation. Breaks in between blocks were self-paced but could not exceed 60 s.

After completing baseline assessments, patients were randomly assigned to the stimulation arms by a computer-generated list (i.e. 32 codes assigning participants to specific experimental protocols, including tDCS polarity, order and right/left coding of response buttons for the experimental task) and participated in the experimental cross-over phase of the study (Fig. 1A). Healthy controls received the same stimulation protocol as patients they were matched to. Two pwRMS in the anodal tDCS arm had to be excluded due to technical errors (i.e. software issues that prevented starting the experimental paradigm) during one of their stimulation sessions.

### Experimental SDMT

**Figure 2:**
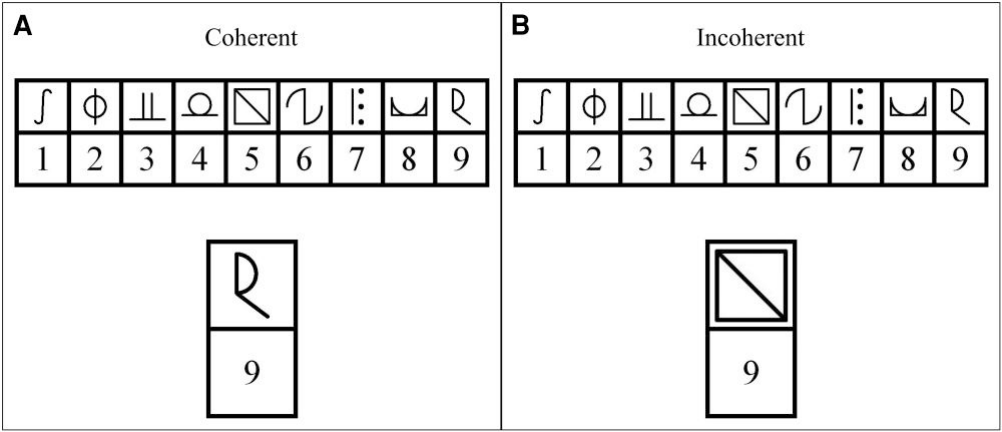
**Experimental task: examples of (A) coherent and (B) incoherent trials of the modified SDMT.** At the top of the image the legend of the current block is displayed, which indicates correct symbol–digit combinations. At the bottom, a ‘probe’ combination is shown and participants had to indicate coherent or incoherent combinations by button press.

To minimize reliance on fine-motor skills required by the original paper-and-pencil SDMT, we developed a computerized version that resembled previous MRI adaptations with a forced-choice button press response mode.^34,45^ This also allowed creating a longer version of the task to enhance statistical power to detect potential stimulation effects. The experimental SDMT paradigm was presented using NBS Presentation® (Version 19.0, Neurobehavioral Systems, Inc., Berkeley, CA, USA). During the experimental sessions, participants completed nine experimental blocks, each with a fixed duration of 90 s (Fig. 1B). Each block comprised nine unique and visually distinct symbols and digits (1–9) and a legend showing nine symbol–digit combinations (Fig. 2A and B). During each trial, the legend was shown at the top of the screen; at the bottom of the screen, a single symbol–digit combination was displayed (probe), which was either coherent or incoherent with the legend. Participants were asked to indicate coherence of the probe with the legend by button presses with left and right index finger (‘M’ and ‘Y’ keys) on a QWERTZ keyboard. Incoherent probes within blocks were created by systematically shifting the symbol–digit combinations. For each coherent combination, eight unique shifted ‘incoherent’ combinations were created (i.e. symbol and digit + 1–8). During each task block, one shifted set was randomly chosen, and coherent and incoherent symbol–digit combinations were pseudo-randomly presented, so that the same digit or symbol was not presented twice in a row. After all pairings were presented, another shifted set was randomly chosen. This assured that approximately the same number of coherent and incoherent trials was presented. The task was self-paced, and the next trial appeared immediately after each response. Participants were instructed to respond as quickly as possible, without making mistakes. No immediate feedback on performance was provided during or after each block. The maximum number of trials per block was 144, which was unattainable within the 90 s block duration. Short breaks were interspersed in between blocks (max. 60 s), and participants could proceed with the next block at their own pace during this time.

### Transcranial direct current stimulation

**Figure 3:**
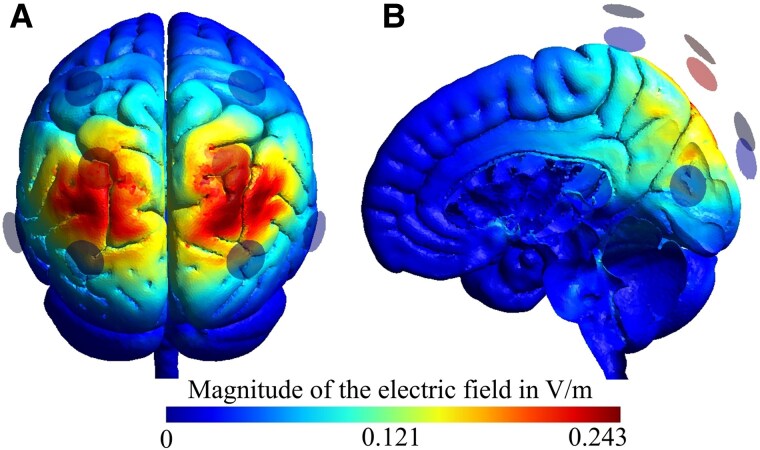
**Current flow simulations bilateral SPL-tDCS.** (**A**) shows peak current intensity in the target region (bilateral superior parietal cortex) and (**B**) medial view illustrates current intensities to deeper brain regions. Note: Standard Simulation of Non-invasive Brain Stimulation (SimNIBS) parameters and a Montreal Neurological Institute 152 standard brain were used for simulations. Current flow patterns are shown for the anodal tDCS condition; cathodal stimulation has a reversed polarity but identical current distribution.

Stimulation was administered with a Neuroelectrics Starstim8 direct current stimulator using Pistim electrodes (radius = 1 cm) and a 3 × 1 setup (i.e. a central active electrode, and three surrounding reference electrodes). Circular inserts in an EEG-cap secured the electrodes on the head. Centre electrodes were positioned bilaterally over the SPL based on 10–10 EEG coordinates (i.e. P1/P2). Return electrodes were positioned in a circular way around the anodes (CP2, P5, PO4, CP1, P6, PO3). This setup was chosen during the planning stage of the project based on current flow simulations using Standard Simulation of Non-invasive Brain Stimulation parameters and a Montreal Neurological Institute 152 standard brain that demonstrated peak current intensities in the superior parietal cortex (Brodmann Area 7) and additional current flow to surrounding and deeper brain regions, including the precuneus (Fig. 3).^46,47^ Stimulation commenced briefly prior to the start of the experimental sessions and was applied with 1.5 mA for 20 min with a 20 s ramp-up and ramp-down at the beginning and the end (active tDCS), or was ramped-up and ramped-down after 40 s (sham tDCS). This procedure has been shown to ensure participant blinding for focal setups.^37,39,41^ Staff administering the stimulation were blinded using predefined stimulation codes that concealed the applied stimulation type. The order of sham and active (i.e. anodal or cathodal) stimulation was counterbalanced, i.e. 50% of the participants received either active or sham tDCS during the first of the two sessions. A minimum of 1 week was maintained between sessions to prevent carry-over effects in the group that received active tDCS first.

An adverse effects questionnaire was completed by the participants after each stimulation session.^13^ Potential unspecific effects of tDCS were assessed with the Positive and Negative Affect Schedule (PANAS) before and after each session.^48^ After the second experimental session, subjects were asked if they believed that active stimulation was administered during the first or the second tDCS session to assess blinding integrity. Potential responses included ‘I do not know’, ‘During the first session’ or ‘During the second session’.

### Outcome measures

Response latency for correct responses was our predefined primary outcome measure (NCT04667221). We also inspected potential effects of tDCS on accuracy as secondary outcome. Response latency data were preprocessed in three steps: (i) all trials with incorrect responses were excluded; (ii) only trials with response latencies between 0.2 and 6 s were included, in line with the upper limit that was used by previous studies that used similar designs^49-51^ and (iii) trials with response latencies within the interval ‘median ± 3×median absolute deviation’, computed on a subject and session basis, were included in the analysis. This approach is more robust than intervals based on *M* values and SDs.^52^ Across sessions and stimulation groups, 5.65–7.06% of trials were excluded (Supplementary Table 1).

### Statistical analyses

Statistical analyses were conducted in a Bayesian framework using R and the *brms* package.^53,54^ Data were analysed using generalized linear mixed models (GLMMs) using a logit-link function for response accuracy, and a lognormal link function for response latency analyses for binomial and skewed data distributions, respectively. The response latency model was truncated to match the range of possible response time outcomes fixed by our filtering procedure. Three models were fitted for each analyses to find the best fitting model for our data: (i) an intercept-only model; (ii) a covariates model to correct for potential session effects, motor slowing via the trail making test (TMT)-A and baseline performance on the paper–pencil SDMT^50^ and (iii) a full model comprising the covariates as specified above, and variables of interest [i.e. stimulation polarity (anodal, cathodal), group (pwRMS, controls) and stimulation type (active, sham)], as well as their interactions. All models included a group-level subject intercept (random intercept) to correct for individual performance levels. To establish the models that best explained our data, models were compared using the widely applicable information criterion (WAIC), which is a fully Bayesian extension of the Akaike information criterion, and the estimated log pointwise predictive density of the WAIC (ELPD WAIC). Difference between ELPD WAIC scores larger than 1.96 times than the standard error of the ELPD difference have been suggested to be meaningful.^55^ For each model, 3000 samples per chain with eight chains were drawn after warm-up, resulting in overall 24 000 draws using the Hamiltonian Monte Carlo Algorithm.^56^ Due to convergence issues, low effective sampling size and high Rhat-values, reaction time models had to be run with 5000 samples after warm-up with eight chains, i.e. 40 000 draws. Furthermore, the random intercept of the intercept model required a soft sum to zero constraint to converge.^57^

An additional exploratory analysis investigated if the degree of IPS impairment in pwRMS (quantified by the continuous baseline SDMT scores) was associated with stimulation response (i.e. reduced or increased response latency in the respective tDCS conditions). For that, the full reaction time model was extended: Baseline performance on the paper–pencil SDMT, group, stimulation polarity and stimulation type, as well as their interactions were added as population-level effects. Additionally, we corrected for TMT-A performance and the session effect.^50^ Subjects were added as group-level intercepts. To ensure the validity of this analysis and enhance clinical relevance of potential outcomes, we considered two different predictors: (i) study-specific raw scores of the baseline SDMT in our sample and (ii) age- and education-corrected norms of the SDMT.^34^

To analyse self-reported adverse effects and PANAS data, we built two GLMMs for each outcome: (i) a simple model that assumed that the effect of active stimulation compared to sham is the same in all experimental groups and (ii) a more complex model that allowed the effect of active stimulation to be distinct, which allows modelling population-specific vulnerability to adverse effects.

For the PANAS data, negative and positive items were summed to build a sum score for each dimension (i.e. positive or negative valence). We fitted a hurdle Gaussian model, since negative affect included many sum scores of 0, but responses were otherwise normally distributed.^58^ The full model included factors for stimulation polarity, group, stimulation type, time point (pre-, post-tDCS) and valence (positive, negative), as well as all interactions. The simple model, however, only included interactions between stimulation type, subject group, time point and valence, and the interaction between active stimulation and time point. Additionally, we modelled the hurdle parameter separately for positive and negative affect, since zeros were only found in the measures of negative affect. The PANAS models included a group-level intercept for subjects.

Adverse effects data were modelled with a cumulative model assuming a continuous latent variable (strength of adverse effect) that was measured using categorical responses (‘none’, ‘mild’, ‘moderate’, ‘strong’).^59^ The full model included stimulation polarity, stimulation type and group, as well as all their interactions. The simple model only considered interactions between stimulation polarity and group. Both models included a population-level factor for sensation types (i.e. itching, pain, burning, heat, metallic taste, fatigue, other) and group-level intercepts for sensation types and subjects.

To investigate blinding of participants, we used categorical models to interrogate the participants’ responses to the question: ‘During which session do you think you were stimulated?’ With categorical models, intercepts represent the shift from one category to another, e.g. the transition from the answer ‘do not know’ to ‘I think, I was stimulated during the first session’. Factors in the models shift the location of these transitions, so that more participants fall into a certain category. Again, we fitted multiple models to describe the data. A full model that included the session when stimulation was applied the group and the stimulation polarity, as well as their interactions. The simple model only included the interaction of group and the stimulation polarity, and a simple effect for the active stimulation session. Thus, we were able to model if the correct response is associated with the participants’ response and consistency of this relationship across groups. Model comparisons for the adverse effects, PANAS, and blinding models were investigated with WAIC and ELPD WAIC as criteria for model comparisons.

### Prior choices

In a Bayesian framework, priors must be chosen to represent expected values that the model parameters may take before the current data were considered. Experimental data shift these priors to represent the expectations after considering data. Priors were chosen to be weakly informative, i.e. population-level parameters were normally distributed with an *M* of 0 and an SD of 1, and group-level standard deviations were set to an exponential distribution with rate of 2 in all models. Note that the blinding assessment did not include group-level intercept, since only one answer per participant was collected.

### Hypothesis testing

Bayesian hypotheses testing used evidence ratios. For directed hypothesis, an evidence ratio is the number of draws in the direction of an effect relative to the number of draws in the opposite direction, i.e. the evidence ratio for a learning effect across session would be computed: evidence  ratio=effectofsession>0/effectofsession<0. Thus, evidence ratios indicate the amount of evidence in favour of a hypothesis compared to the direct counter hypothesis. Evidence ratios were computed for the model parameters and *post hoc* comparisons. The theoretical range of evidence ratios is 0 to ∞, whereby evidence ratios of ∞ indicate that all posterior draws were in favour of the tested hypotheses. For point hypotheses, i.e. tests for equality, evidence ratios indicate an increase or decrease of evidence compared to the prior model.^54^ Simulations with linear and logistic regression models have found that evidence ratios of 19 and 39 correspond to one- and two-sided hypothesis tests with an alpha-level of 0.05.^60^ Note that we are unaware of formal comparisons between evidence ratios and *P*-values in GLMMs. *Post hoc* comparisons were always comprised of within-subject comparisons of active session to the respective sham sessions. For accuracy and response latency models, differences between the sessions were tested, while adverse effects, blinding, and PANAS models tested equality of sessions.

## Results

Overall, participants tolerated the stimulation well, there was no evidence that potential changes in mood and affect biased the behavioural tDCS effects and participant blinding was successful (see below for details).

### Accuracy

Response accuracy was near ceiling level across groups (*M* = 98.23%, SD = 13.2%); group means are shown in Supplementary Table 2, indicating compliance to the instructions to answer as fast as possible without making mistakes. WAIC revealed that the intercept-only model performed best (Supplementary Table 3). However, this suggests that the factors of interest, i.e. group, stimulation type and stimulation polarity, as well as their interactions, and covariates did not increase the model fit (Supplementary Table 4).

### Response latency

Assessment of the response latency models showed that the full model including covariates and the effects of interest performed best, indicated by lower ELPD WAIC and WAIC values (Supplementary Table 5).

**Figure 4:**
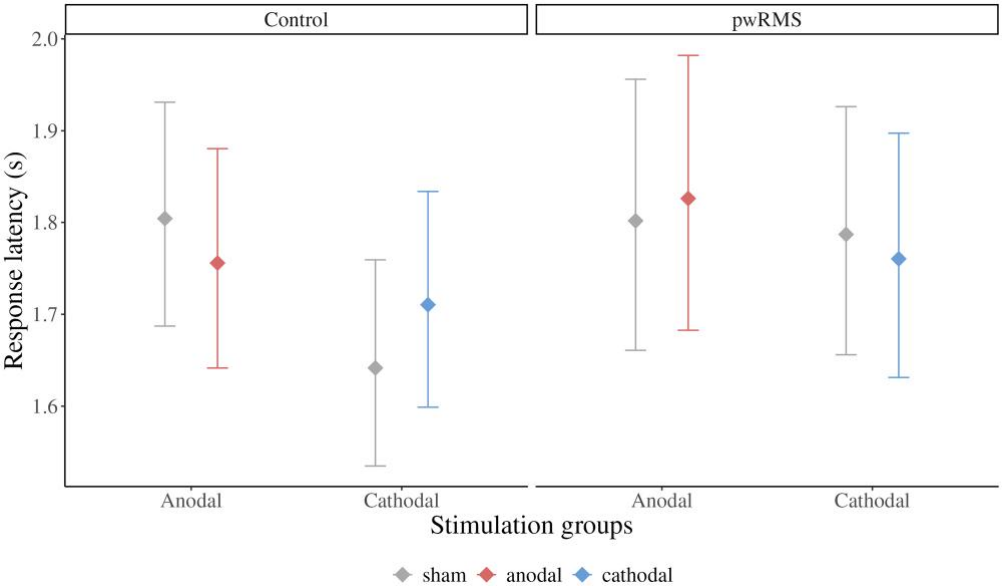
**Response latency effects.** Conditional effects plot based on a GLMM with lognormal link function. Response latency effects of anodal and cathodal stimulation in both subject groups, controlled for session effects, motor slowing (via TMT-A), and the number of correct answers in the paper–pencil SDMT. Compared to sham stimulation, healthy controls exhibited shorter response latencies during anodal stimulation and increased response latencies during cathodal stimulation. Across the groups, this effect was reversed in patients with RMS, i.e. response latencies were shorter during cathodal stimulation and longer during anodal stimulation (relative to sham stimulation; *β* = −0.10, 95% CI = −0.11 to −0.08, evidence ratio *=* ∞, *N*_subjects_ = 62, *N*_observations_ = 52 764).

The full model for response latency provides strong evidence for a three-way interaction between the effects of interest: group × stimulation type × polarity [*β* = −0.10, 95% confidence interval (CI) = −0.11 to −0.08, evidence ratio *=* ∞; Fig. 4]. In the control group, *post hoc* tests showed that anodal stimulation decreased reaction times (*β*  *=* −0.03, 95% CI = −0.03 to −0.02, evidence ratio = ∞) and cathodal stimulation increased reaction times (*β* = 0.04, 95% CI = 0.04–0.05, evidence ratio = ∞) compared to their respective sham conditions. However, in the patients, cathodal stimulation decreased response latencies (*β* = −0.02, 95% CI = −0.02 to −0.01, evidence ratio *=* 4443.44), while anodal stimulation increased response latencies (*β* = 0.01, 95% CI = 0.01–0.02, evidence ratio = 929.23) relative to sham tDCS. Hence, we demonstrate a double dissociation for effects of anodal versus cathodal tDCS in pwRMS and healthy controls (Supplementary Table 6).

Additionally, there was strong evidence that the covariates included in our model influenced latency in the modified SDMT. Participants were faster during the second session suggesting a learning effect (*β* = −0.12, 95% CI = −0.12 to −0.11, evidence ratio *=* ∞). Longer response latencies in the TMT-A were positively correlated with longer response times in the experimental SDMT (*β* = 0.06, 95% CI = 0.01–0.10, evidence ratio = 129.72), i.e. the term corrected for motor slowing. Additionally, an increased number of correct responses in the baseline paper–pencil SDMT, i.e. an indirect measure of shorter response latencies during the baseline SDMT, was associated with shorter response latencies in the modified SDMT (*β* = −0.17, 95% CI = −0.21 to −0.12, evidence ratio *=* ∞). Therefore, this covariate corrected for group differences in the baseline SDMT performance and the results provide strong evidence for a correspondence between the clinical and experimental versions of the SDMT.

### Relation between baseline IPS performance and tDCS response

Results were comparable for the study-specific raw scores of baseline SDMT performance and the additional analysis that used age-corrected norms.^34^ Model comparison between the study-specific exploratory model and the full model from the previous analysis yielded better ELPD WAIC scores for the exploratory model (ELPD difference = −255.27; SE difference = 25.12; |−255.27| > 49.23).

**Figure 5:**
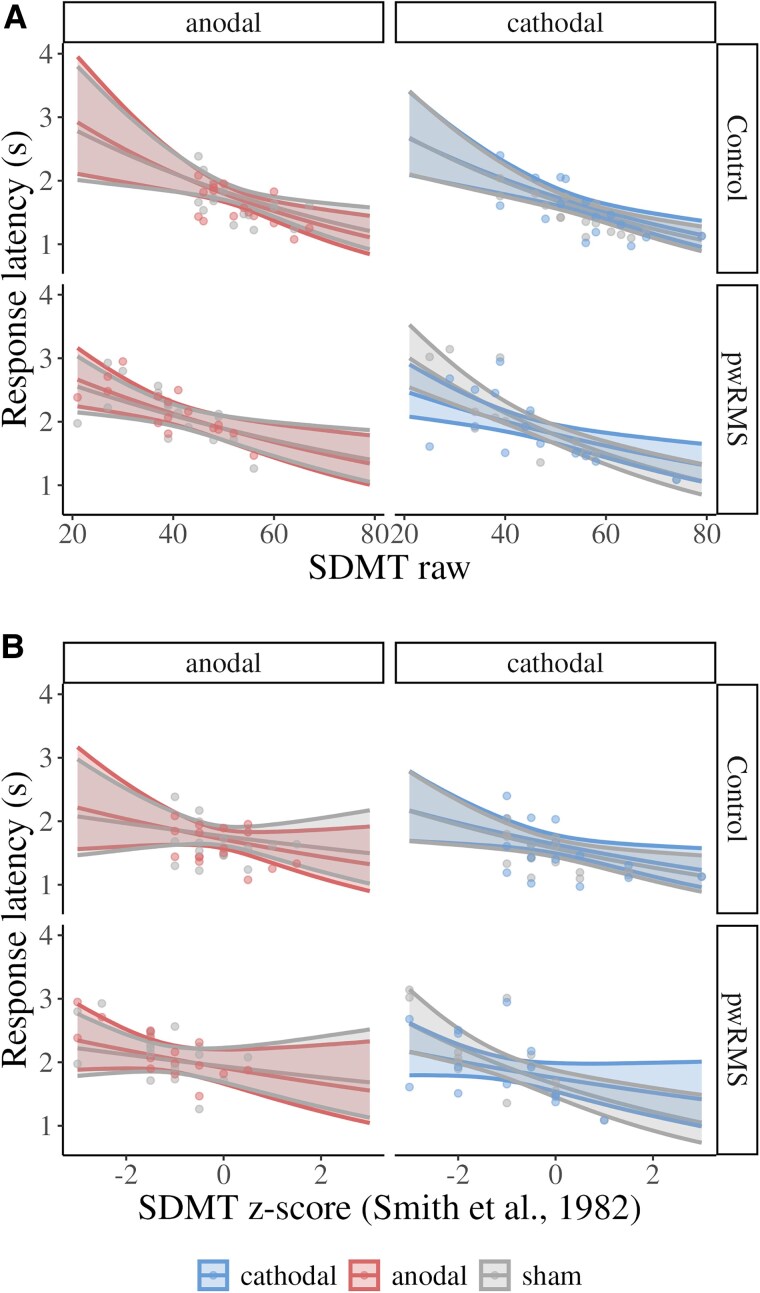
**Conditional effects plot.** Based on GLMMs with lognormal link function. The three-way interactions for the association between response latency during either placebo (sham) and anodal or cathodal tDCS are illustrated. (**A**) Study-specific distribution of raw scores of the baseline SDMT (*β* = 0.06, 95% CI = 0.04–0.08, evidence ratio *=* ∞, *N*_subjects_ = 62, *N*_observations_ = 52 764). (**B**) Age-corrected SDMT norms (*β* = 0.06, 95% CI = 0.04–0.08, evidence ratio *=* ∞, *N*_subjects_ = 62, *N*_observations_ = 52 764). Controls did not reach *z*-scores less than −1 indicating that the patient group drives the reversal of the AeCi response. The *x*-axis shows the raw or *z*-scores of the baseline. Note the raw SDMT scores were standardized during model computation, but rescaled for plotting (*M* = 48.15, SD = 11.93). The *y*-axis shows mean response latency during sham or active (anodal, cathodal) tDCS conditions. Ribbons represent a 95% credible interval. Dots represent mean response latency of individual participants across experimental blocks and baseline SDMT scores of the participants.

A four-way interaction between group, polarity, stimulation type and baseline SDMT score was confirmed for the study-specific and norm-based analyses (study specific: *β* = 0.06, 95% CI = 0.04–0.08, evidence ratio *=* ∞; age-corrected norms: *β* = 0.06, 95% CI = 0.04–0.08, evidence ratio *=* ∞; Fig. 5). This interaction has to be interpreted with caution because the group factor and baseline SDMT scores are related, i.e. patients generally have lower SDMT scores than healthy controls. The latter is illustrated in the upper panels of Fig. 5B (i.e. lack of *z*-scores lower than −1 in healthy controls). Nonetheless, healthy controls with higher baseline SDMT scores showed a stronger canonical AeCi response to tDCS, i.e. anodal stimulation reduced response latency and cathodal stimulation increased response latency, relative to sham stimulation. Because none of the healthy controls had *z*-scores lower than −1, only the canonical response was observed.

However, the four-way interaction also indicates that the moderating effect of baseline SDMT was larger in pwRMS. In addition, since we included patients with MS that had *z*-scores lower than −1, we observed a reversal of the canonical AeCi response, i.e. cathodal reduced response latency and anodal increased response latency (Supplementary Tables 7 and 8). Notably, for standardized scores, the transition from beneficial to non-beneficial effects of anodal (*z* < −0.58) or cathodal (*z* > −0.70) was consistent across the patient groups.

We additionally repeated the analyses and only included the patient subgroups to investigate if the control group drove the interaction effect. Here, we found a three-way interaction of stimulation polarity, stimulation type and baseline SDMT score, indicating that this was not the case (Supplementary Tables 9 and 10).

### Adverse effects, PANAS and blinding

Self-reported adverse effects were mainly rated as ‘none’ (81.73%) or ‘mild’ (14.05%). ‘Moderate’ and ‘strong’ adverse effects were only reported in 0.6–3.63% of the participants (Table 2). Model comparison of adverse effects models favoured the simple model assuming that the strength of adverse effects is similar in all experimental groups since the absolute ELPD difference was smaller than the standard error multiplied by 1.96 (ELPD difference = −2.4; SE difference = 2.9; |−2.4| < 5.68). A *post hoc* test showed a beta value for stimulation around 0, and a small increase of evidence from the prior model (*β* = 0.03, 95% CI = −0.18 to 0.23, evidence ratio = 9.44), suggesting that adverse effects were comparable between active and sham conditions (see Supplementary Table 11).

**Table 2:** Mean adverse effects score, when assigning 0–3 to none, mild, moderate and strong adverse effects

Sensation	Healthy controls								pwRMS							
	Anodal				Cathodal				Anodal				Cathodal			
	Sham		Active		Sham		Active		Sham		active		Sham		Active	
	*M*	SD	*M*	SD	*M*	SD	*M*	SD	*M*	SD	*M*	SD	*M*	SD	*M*	SD
Heat	0.25	0.45	0.50	0.63	0.19	0.40	0.12	0.50	0.14	0.53	0.06	0.25	0.07	0.27	0.21	0.58
Itching	0.25	0.45	0.31	0.48	0.56	0.73	0.25	0.45	0.00	0.00	0.31	0.60	0.14	0.53	0.21	0.58
Burning	0.19	0.40	0.50	0.63	0.69	0.70	0.38	0.50	0.00	0.00	0.25	0.58	0.07	0.27	0.21	0.58
Pain	0.19	0.54	0.19	0.40	0.19	0.54	0.06	0.25	0.07	0.27	0.06	0.25	0.21	0.43	0.29	0.61
Metallic taste	0.00	0.00	0.00	0.00	0.00	0.00	0.00	0.00	0.00	0.00	0.00	0.00	0.00	0.00	0.00	0.00
Fatigue	0.44	0.81	0.19	0.54	0.31	0.48	0.25	0.45	0.43	0.85	0.38	0.62	0.43	0.76	0.43	0.94
Other	0.19	0.40	0.06	0.25	0.19	0.40	0.00	0.00	0.43	0.65	0.75	1.00	0.64	1.01	0.64	0.84

Comparison of the PANAS models also favoured the simpler model, since the full model only yielded marginally better model criteria (ELPD difference = −0.5; SE difference = 2.5; |−0.5| < 4.9). This suggests that PANAS change scores (i.e. pre- to post-active versus sham tDCS) were comparable in all experimental groups. *Post hoc* tests showed weak evidence that the reported affect changes from pre- to post-stimulation is the same during sham and active stimulation in all groups (*β* = 0.21, 95% CI = −1.11 to 1.55, evidence ratio = 1.45). The full model and summary statistics (groups, valence) are reported in Supplementary Tables 12 and 13.

Assessment of blinding shows that many participants reported to either ‘not know’ when they were stimulated (41.94%) or stimulated during the second session (37.10%); the latter suggests a recency effect. Model comparisons between the three models showed that the full model performed slightly better than the simple model (ELPD difference = −3.3; SE difference = 1.4; |−3.3| > 2.81). The full model, however, did not show a clear pattern for the response behaviour of participants. Most pronounced evidence was found for a different response pattern in pwRMS. They were more likely to answer that they did not know when they were stimulated and less likely to answer that they think they were stimulated during the second session (*β* = −0.91, 95% CI = −2.12 to 0.28, evidence ratio = 13.32; Supplementary Fig. 1A). Similarly, people receiving cathodal stimulation during the second session were more likely to report that they did not know when they were stimulated and less likely to report that they thought they were stimulated during the second session (*β* = −1.09, 95% CI = −2.52 to 0.36, evidence ratio = 13.00; Supplementary Fig. 1B). However, the posterior distributions for all predictors were relatively wide, which does not allow for a clear interpretation of effects. Hence, it is concluded that blinding of participants was successful. For details, see Table 3 and Supplementary Table 14.

**Table 3:** Counts for responses to the question: ‘When do you think you were stimulated?’

Group	Stimulation polarity	Active Session	Do not know	Session 1	Session 2
Control	Anodal	Session 1	2	3^a^	3
		Session 2	3	1	4^a^
	Cathodal	Session 1	0	1^a^	7
		Session 2	4	4	0^a^
People with MS	Anodal	Session 1	3	3^a^	2
		Session 2	5	0	3^a^
	Cathodal	Session 1	5	0^a^	1
		Session 2	4	1	3^a^

^a^The session in which active stimulation was administered.

## Discussion

This study presents strong evidence for causal involvement of bilateral SPL in the speed component of IPS in health and disease. Moreover, by identifying a clinically relevant participant-dependent predictor of stimulation response in pwRMS (i.e. clinical SDMT performance), we provide a framework for future individualized neurostimulation treatments of impaired IPS in this population. Our main findings can be summarized as follows:

1. Relative to sham tDCS, healthy individuals showed the expected polarity-specific pattern of increased (cathodal tDCS) or decreased (anodal tDCS) response latency during the experimental SDMT, which provides strong causal evidence for involvement of bilateral SPL in healthy individuals.
2. At the group level, the opposite pattern was found in the patient groups, and there was strong evidence for a double dissociation of tDCS effects between pwRMS and age- and sex-matched healthy controls. This emphasizes that stimulation effects observed in healthy individuals may not necessarily translate in a one-by-one fashion to clinical populations with structural and functional brain reorganization.
3. However, our results also demonstrate that the degree of baseline IPS impairment in the patient groups mediated stimulation response in a polarity-specific way. While more impaired patients responded favourably to cathodal tDCS (transition at *z* = −0.70), less impaired patients improved more when receiving anodal tDCS (transition *z* = −0.58). This result is in line with functional imaging studies in pwRMS (for review see Roca *et al*.^28^), which have suggested compensatory upregulation of metabolic brain activity in task-relevant brain networks in patients with less pronounced motor or cognitive impairment. In more impaired patients, enhanced activity may indicate dysfunctional processes, involving the breakdown of functional network communication or disinhibition. Notably, the predictive value of baseline SDMT impairment for stimulation response in pwRMS was confirmed for both the study-specific distribution of baseline SDMT values and standard scores, which will be particularly valuable to inform clinical tDCS treatment decisions in the future.^34^
4. Finally, the rigorous design of our study and the high level of experimental control ensured that our results cannot be explained by unspecific factors (e.g. unblinding of participants or staff, tDCS effects on mood or affect, order effects in the cross-over phase). In addition, adverse effects were minimal and comparable between groups and stimulation conditions, which highlight safety, tolerability and feasibility of our focalized tDCS approach.

Over the last two decades, tDCS has been studied extensively for its potential to improve human brain function. The majority of this research has focused on motor functions, which has also been a major focus in pw(R)MS.^20,21^ However, there is increased evidence that higher cognitive functions that are known to be supported by large-scale neural networks^61^ can be improved by tDCS. Notably, stimulation administered to key nodes of task-relevant cognitive networks has not only shown promise to enhance performance and brain function in healthy individuals and neuropsychiatric diseases^14,62^ but also revealed substantial variability of tDCS response within and across studies. The underlying sources of this variability are thought to be multifactorial and can broadly be described as participant-dependent (e.g. skull and brain anatomy or functional network organization) and stimulation-dependent factors (e.g. tDCS timing, duration, intensity or focality), as well as their interactions.^12,63^ Moreover, this research has highlighted that structural and functional brain organization due to normal ageing or in patients with neuropsychiatric diseases can alter stimulation response relative to healthy young or age-matched reference populations, respectively.^15,62^ This emphasizes the necessity to conduct carefully designed proof-of-concept studies to investigate the effects of specific tDCS protocols in clinical populations and to identify predictors of stimulation response, prior to implementation in time- and cost-intensive clinical trials. This is particularly relevant in pwRMS, where less than a handful of studies have investigated potential tDCS effects on cognition and yielded mixed results.^20,21^

Consequently, the present study implemented a highly controlled, prospective, randomized, triple-blinded, balanced cross-over trial that involved the assessment of polarity effects and employed a focalized tDCS setup, aimed at confirming the causal role of the SPL in IPS in pwRMS and matched healthy controls. The relevance of the stimulation target was informed by imaging studies in pwRMS that suggested involvement of bilateral SPL in the speed component of IPS, functional magnetic resonance imaging (fMRI) adaptations of the SDMT resembling the task used in the present study and also evidence from lesion studies demonstrating specific involvement of SPL in the manipulation and re-arrangement of information in working memory, which is highly relevant for the SDMT.^35,36,45,64^ Moreover, the adapted experimental paradigm was designed to minimize dependence on fine-motor skills compared to the clinically used paper-and-pencil version and allowed increasing the number of trials to enhance statistical power to detect potential tDCS effects.^34^ Our results also provide strong evidence for a correspondence between the novel computerized and paper–pencil versions of the SDMT, which highlights construct validity of the experimental paradigm employed in this study. Using this task and overall research design, we were able to confirm causal involvement of the SPL in SDMT performance in healthy controls and pwRMS. In addition, we identified a clinically relevant predictor of stimulation response in the patient groups (clinical SDMT impairment) that may be valuable to guide future tDCS treatment studies.

The canonical AeCi response in healthy individuals is in line with the proposed up- and downregulation of neural excitability frequently observed in the motor system.^65^ However, polarity-specific modulation of behavioural performance has been less consistent for cognition. This is likely explained by higher redundancy within the neural networks supporting cognitive functions, rendering them less malleable to effects of cathodal tDCS.^66^ Hence, our results contribute to the ongoing debate if tDCS can modulate cognition in a polarity-specific way and strengthens the assumption that bilateral SPL is critically involved in IPS in healthy individuals.

In pwRMS, Bayesian modelling provided strong evidence for a double dissociation of tDCS effects relative to healthy controls, which emphasizes that stimulation effects in pwRMS cannot easily be derived from outcomes in neurotypical individuals. Most strikingly, however, patients with more pronounced impairment responded more favourably to cathodal tDCS, while less impaired patients showed a beneficial response to anodal tDCS. Overall, this polarity by impairment interaction in pwRMS is in line with imaging studies, suggesting a gradient of neural compensation and dysfunction that depends on the level of impairment in specific tasks.^28-33^ Notably, similar mechanisms have been suggested for normal ageing, where increased task-related activity in frontoparietal regions can effectively compensate for structural and functional brain changes to a certain degree of task difficulty, while this may not be possible with more pronounced neurodegeneration.^67,68^ Hence, facilitation of compensatory processes by anodal tDCS in earlier stages of the disease and inhibition of dysfunctional processes later (e.g. increased neural noise due to reduced lateral inhibition or disconnection) are likely candidates to explain our findings in pwRMS. Importantly, our sample was comprised of mild to moderately impaired patients, suggesting that the transition between the two processes may happen relatively early. This is also in line with one of the few longitudinal fMRI studies in pwRMS that demonstrated an association of increased parietal activity over time with SDMT performance declines.^69^ However, in the future, this hypothesis could be investigated more directly by administering bilateral focal SPL-tDCS concurrently with fMRI adaptation of the SDMT to confirm the proposed mechanisms.^12,70^

### Limitations

First, our sample size was relatively small, only including pwRMS (excluding patients with progressive MS), and for feasibility reasons, we only recruited patients with mild to moderate levels of functional impairment, thereby limiting the generalizability of the results. Nonetheless, even in this sample, we observed a statistically sound interaction between group, polarity and stimulation condition and a dissociation between effects of anodal and cathodal tDCS that was explained by baseline SDMT impairment. While this suggests that patients with higher levels of impairment and brain dysfunction may benefit from cathodal SPL-tDCS, this needs to be confirmed in the future. Moreover, the double dissociation of tDCS effects between healthy individuals and pwRMS likely reflects study-specific distributions of (mild to moderate) baseline impairment in the patients and may not generalize to samples including more impaired patients. Nonetheless, the impairment profile of patients recruited for this study highlights that the transition from compensatory to dysfunctional activity in the SPL may happen relatively early in the course of the disease and is therefore suited to inform future tDCS treatment decisions.

Second, because our proof-of-principle study did not involve concurrent fMRI, the proposed mechanisms by which anodal or cathodal tDCS improved SDMT performance remain to be determined. Importantly, the experimental SDMT used in this study was designed to require minimal adaptations for future tDCS-fMRI implementations, to investigate the neural mechanisms of impairment (during sham tDCS) and neuromodulation by active tDCS (anodal versus cathodal).

Third, based on the limited number of studies that investigated tDCS effects on cognition in pwRMS and to prevent inducing maladaptive neuroplastic adaptation, we opted for a cross-over design with a single active tDCS session. Because effects of single tDCS sessions are transient and reversible, this assured that no long-term negative effects would be induced.^65^ Notably, more pronounced and longer lasting tDCS effects have been confirmed for learning compared to cognitive tasks and for multisession tDCS in combination with behavioural training or therapy.^26,71-74^ In this context, it is acknowledged that our study only provides proof-of-principle evidence and we abstain from claiming that the observed effects are clinically relevant. Nonetheless, the direction of effects is plausible regarding imaging findings in pwRMS and theoretical accounts of tDCS effects. In addition, the reported evidence ratios for our primary outcome (response latency) ranged from 129.72 to ∞. Evidence ratios can roughly be ‘translated’ into corresponding *P*-values used in traditional frequentist statistics frameworks and the theoretically corresponding *P*-value range would be between 0.008 [1/(129.72 + 1)] and <0.000025 [<1/(40 000 + 1)].^60^ Thus, our results provide a theoretically and statistically sound rationale and guidance for future intervention studies that combine traditional neurocognitive or gaming-based interventions aimed at improving IPS with tDCS, which may be suited to induce more pronounced and lasting effects on IPS than those observed in the present study.^75,76^

Fourth, we did not formally assess the impact of fatigue/fatigability on tDCS effects. While this may be more relevant for cross-sectional studies investigating cognitive impairment in pwRMS, future cross-over tDCS studies are advised to assess fatigue levels prior to and after each experimental session to formally investigate the potential impact of this factor. Finally, using alternate versions of the experimental paradigm may have reduced the learning effects observed in this study (even though those were controlled for in our statistical analyses).

## Conclusion

We provide strong evidence for causal involvement of SPL in IPS in health and disease. The double dissociation of tDCS effects in pwRMS and healthy controls and variability of stimulation effects within the patient groups highlights the need to tailor stimulation protocols based in relevant participant-dependent factors. Our own data suggest that performance on the clinical version of the SDMT may be suited to inform tDCS treatment decisions in pwRMS and that the degree of IPS impairment reflects compensatory or dysfunctional neuroplastic processes in the SPL, which can be modulated by tDCS in a polarity-specific way and that the transition between compensation and dysfunctional breakdown of neural networks supporting cognition may happen early in the course of disease. Our findings will help inform future clinical trials aimed at improving impaired IPS in a more sustained way by combining repeated tDCS sessions and behavioural interventions.

## Supplementary Material

fcaf223_Supplementary_Data

## Data Availability

The experimental paradigm and data as well as scripts for data analyses are available at the Open Science Framework (OSF).
