# Supplementary material for: Information processing speed modulation by electrical brain stimulation in multiple sclerosis: towards individually tailored protocols
Source: Brain Commun. 2025 Jun 6;7(3):fcaf223. doi: 10.1093/braincomms/fcaf223 (PMC12198773; doi:10.1093/braincomms/fcaf223)
Supplement: fcaf223_Supplementary_Data [file fcaf223_supplementary_data.pdf]

Supplementary Materials

Supplementary Figure 1. Conditional Effects of the blinding model. Illustration of the effect (A) that patients were more likely to answer that they do not know, when they were stimulated and (B) that people that were stimulated during the second session with cathodal stimulation were more likely to answer that they do not know when they were stimulated.

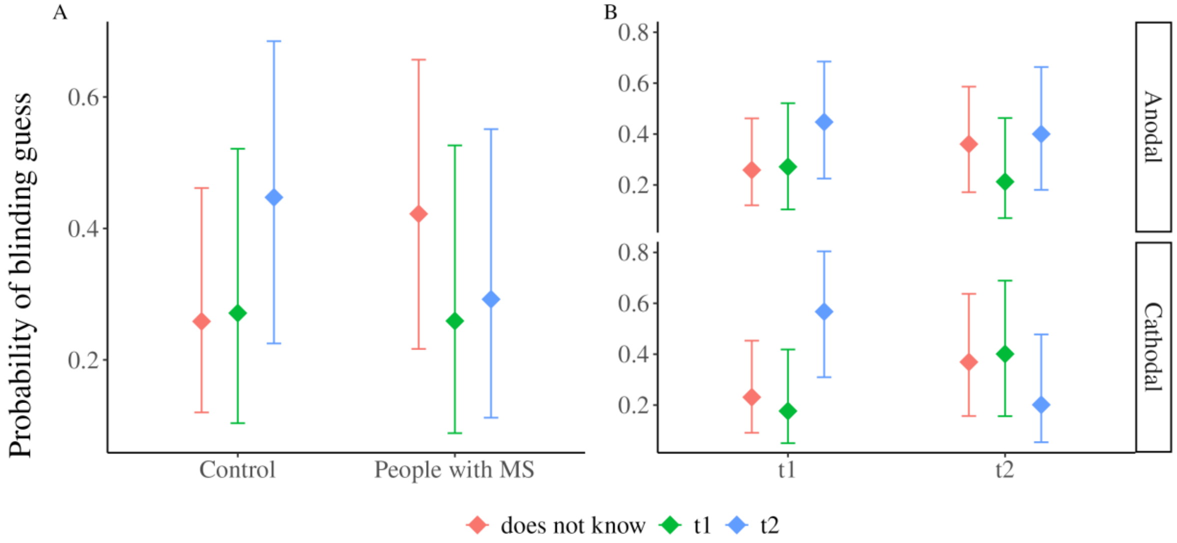

Supplementary Table 1

Absolute number and percentage (relative to all trials after each filtering step) of included trials.

	Control				*MS Patients*			
	anodal		*cathodal*		*anodal*		*cathodal*	
	Sham	*Active*	*Sham*	*Active*	*Sham*	*Active*	*Sham*	*Active*
All trials	7652 (100%)	7878 (100%)	8789 (100%)	8536 (100%)	5918 (100%)	5785 (100%)	5685 (100%)	6021 (100%)
Step (1)	7543 (98.58%)	7743 (98.29%)	8631 (98.2%)	8370 (98.06%)	5776 (97.6%)	5672 (98.05%)	5592 (98.36%)	5937 (98.6%)
Step (2)	7541 (98.55%)	7739 (98.24%)	8628 (98.17%)	8361 (97.95%)	5757 (97.28%)	5652 (97.7%)	5566 (97.91%)	5924 (98.39%)
Step (3)	7220 (94.35%)	7410 (94.06%)	8203 (93.33%)	7972 (93.39%)	5500 (92.94%)	5437 (93.98%)	5344 (94%)	5678 (94.3%)

Note. Data were cleaned in the following order: (1) Removal of incorrect responses, (2) Removal of trials with response times outside the 0.2-6s interval, (3) Removal of trials with response latencies outside of the individual median ± 3 × MAD interval. The individual median was calculated for each subject and session. MAD=Median Absolute Deviation. s=seconds.

Supplementary Table 2

Summary of accuracy data

	Control				*pwRMS*			
	anodal		*cathodal*		*anodal*		*cathodal*	
	M	*SD*	*M*	*SD*	*M*	*SD*	*M*	*SD*
sham	0.99	0.12	0.98	0.13	0.98	0.15	0.98	0.13
active	0.98	0.13	0.98	0.14	0.98	0.14	0.99	0.12

Note. The table shows the mean and standard deviation of accuracy for each group, stimulation group and active condition. M=Mean, SD=Standard deviation. pwRMS=patients with relapsing multiple sclerosis

Supplementary Table 3

WAIC comparison of response accuracy models. ELPD diff shows the difference in the ELPD WAIC-value relative to the best performing model (here: model including covariates).

Model	*ELPD diff*		*ELPD WAIC*		*pWAIC*		*WAIC*	
	Est	*SE*	*Est*	*SE*	*Est*	*SE*	*Est*	*SE*
Covariates model*	0.00	0.00	-4,866.42	122.35	48.76	1.62	9,732.85	244.69
Full model*,#	-1.40	2.48	-4,867.82	122.45	53.40	1.76	9,735.65	244.90
Intercept only model	-8.02	4.14	-4,874.44	122.53	48.99	1.64	9,748.89	245.07

Note. Models are ranked according to their ELPD WAIC score, i.e., the top model reached the best quality scores, while the bottom model has the worst. While the intercept only model does not yield better model criteria (|-8.02| < 8.11), the simpler intercept only model is favored over the more complex covariates model or the full model. ELPD=Expected log pointwise predictive density, SE=Standard error, WAIC=Widely applicable information criterion, pWAIC=Effective number of parameters.

* Includes covariates to correct for the effects of TMT-A time, SDMT n correct at neuropsychology, and session.

# Includes effects of interest, i.e., subject group, active stimulation, and stimulation type, as well as their interactions.

Supplementary Table 4

Population-level effects of the response accuracy model

Parameter	*Estimate*	*SE*	*Rhat*	*Bulk ESS*	*Tail ESS*	*Evidence ratio*	*95% CI*
Intercept	3.98	0.08	1.00	8,990.16	13,398.93	∞*	[3.82, 4.14]
Session	0.25	0.06	1.00	38,603.47	17,243.19	∞*	[0.13, 0.38]
TMT-A time	0.01	0.09	1.00	8,353.86	12,175.39	1.11	[-0.18, 0.19]
SDMT n correct	0.23	0.10	1.00	7,842.09	10,465.31	124.00	[0.04, 0.42]

Note. Subject group-level intercepts Estimate: 0.52 (95%CI [0.41, 0.65]). Rhat: 1, Bulk ESS: 7001.74; Tail ESS: 11749.66. Rhat values should be close to 1 to indicate convergence. Bulk ESS and Tail ESS indicate the effective sample size of the MCMC chains. Evidence ratio indicate the ratio of draws that were in the direction of the estimate relative to the number of draws that were in the opposite direction, e.g., for the TMT-A time effect, there were 1.11 times more positive draws than negative draws. Simulations in linear models show, that an evidence ratio of 19 is equivalent to a p-value of 0.05 (Makowski et al.).[1] SE=Standard error. ESS=Effective sample size. TMT-A=Trail making test version A. SDMT=Symbol-digits-modalities-test.

* Evidence ratios equal to ∞ indicate that all posterior draws were in favor of the estimate direction.

Supplementary Table 5

WAIC comparison of response latency models. ELPD diff shows the difference in the ELPD-value relative to the best performing model (here: the full model).

Model	*ELPD diff*		*ELPD WAIC*		*pWAIC*		*WAIC*	
	Est	*SE*	*Est*	*SE*	*Est*	*SE*	*Est*	*SE*
Full model[*,#]	0.00	0.00	-21,282.99	190.79	70.39	0.48	42,565.98	381.57
Covariates model[#]	-95.72	13.70	-21,378.70	191.43	66.04	0.45	42,757.41	382.86
Intercept only model	-1,751.70	58.11	-23,034.69	194.51	65.07	0.45	46,069.38	389.03

Note. Models are ranked according to their ELPD WAIC score, i.e., the top model reached the best quality scores, while the bottom model has the worst. ELPD WAIC=Expected log pointwise predictive density of WAIC, ELPD diff=difference of the ELPD relative to the best performing model. SE=Standard error, WAIC=Widely applicable information criterion, pWAIC=Effective number of parameters.

[*] Includes effects of interest, i.e., subject group, active stimulation, and stimulation type, as well as their interactions.

[#] Includes covariates to correct for the effects of TMT-A time, SDMT n correct at neuropsychology, and session.

Supplementary Table 6

Population-level effects of the response latency model

Parameter	*β*	*SE*	*Rhat*	*Bulk ESS*	*Tail ESS*	*Evidence ratio*	*95% CI*
Intercept	-0.53	0.06	1.00	5,393.32	8,683.31	∞[1]	[-0.65, -0.40]
Session	-0.20	0.01	1.00	24,131.12	17,560.29	∞[1]	[-0.23, -0.17]
TMT-A time	0.10	0.04	1.00	6,115.02	10,126.79	239.00	[0.03, 0.17]
SDMT n correct	-0.15	0.04	1.00	5,943.17	8,101.04	2,399.00	[-0.23, -0.06]
Group	0.07	0.10	1.00	4,438.55	6,892.26	3.09	[-0.13, 0.27]
Stimulation type	-0.07	0.03	1.00	11,926.56	15,985.20	97.36	[-0.13, -0.01]
Stimulation polarity	-0.03	0.09	1.00	5,197.34	8,273.01	1.82	[-0.21, 0.15]
Group × Stimulation type	0.14	0.04	1.00	11,612.33	15,137.24	4,799.00	[0.07, 0.21]
Group × Stimulation polarity	0.20	0.13	1.00	4,687.82	7,565.96	14.16	[-0.06, 0.44]
Stimulation type × Stimulation polarity	0.19	0.05	1.00	11,221.91	14,820.39	∞*	[0.10, 0.28]
Group × Stimulation type × Stimulation polarity	-0.40	0.06	1.00	11,330.79	15,259.31	∞*	[-0.51, -0.29]

Note. Group-level subject intercepts *SD*=0.24 (95%CI [0.19, 0.29]). Rhat: 1, Bulk ESS: 4997.58; Tail ESS: 8034.29. Rhat values should be close to 1 to indicate convergence. Bulk ESS and Tail ESS indicate the effective sample size of the MCMC chains. Evidence ratios indicate the ratio of draws that were in the direction of the estimate relative to the number of draws that were in the opposite direction, e.g., for the TMT-A time effect, there were 239 times more positive draws than negative draws. Simulations in linear and ordinal regressions show that an evidence ratio of 39 is equivalent to p-value of 0.05 in a two sided hypothesis test (Makowski et al.).[1] SE=Standard error. ESS=Effective sample size. TMT-A=Trail making test version A. SDMT=Symbol-digits-modalities-test.

* Evidence ratios equal to ∞ indicate that all posterior draws were in favor of the estimate direction.

Supplementary Table 7

Population-level effects of the exploratory response latency model.

Parameter	*Estimate*	*SE*	*Rhat*	*Bulk ESS*	*Tail ESS*	*Evidence ratio*	*95% CI*
Intercept	0.62	0.04	1.00	3,179.59	6,025.48	∞*	[0.53, 0.70]
Session	-0.13	0.00	1.00	56,198.99	27,085.32	∞*	[-0.13, -0.12]
TMT-A time	0.06	0.02	1.00	5,221.33	10,224.62	155.86	[0.01, 0.11]
SDMT n correct	-0.17	0.06	1.00	3,668.91	6,422.69	438.56	[-0.29, -0.05]
Group	0.02	0.06	1.00	3,486.25	6,803.91	1.85	[-0.10, 0.15]
Stimulation type	-0.01	0.00	1.00	12,557.31	22,480.76	1,211.12	[-0.02, -0.01]
Stimulation polarity	-0.08	0.06	1.00	3,484.60	6,802.86	10.03	[-0.19, 0.04]
SDMT n correct × Group	0.05	0.07	1.00	3,543.67	7,501.43	2.96	[-0.10, 0.20]
SDMT n correct × Stimulation type	-0.03	0.01	1.00	10,548.24	19,628.86	∞*	[-0.04, -0.02]
Group × Stimulation type	0.02	0.01	1.00	16,297.60	23,575.58	80.14	[0.00, 0.03]
SDMT n correct × Stimulation polarity	-0.02	0.07	1.00	3,897.37	7,173.21	1.48	[-0.16, 0.13]
Group × Stimulation polarity	0.04	0.09	1.00	3,683.45	7,715.94	2.01	[-0.13, 0.20]
Stimulation type × Stimulation polarity	0.05	0.01	1.00	12,506.82	22,130.52	∞*	[0.03, 0.06]
SDMT n correct × Group × Stimulation type	0.01	0.01	1.00	12,172.81	20,597.85	6.88	[-0.01, 0.03]

Supplementary Table 7

Population-level effects of the exploratory response latency model.

Parameter	*Estimate*	*SE*	*Rhat*	*Bulk ESS*	*Tail ESS*	*Evidence ratio*	*95% CI*
SDMT n correct × Group × Stimulation polarity	-0.07	0.09	1.00	3,986.60	8,170.75	3.71	[-0.25, 0.11]
SDMT n correct × Stimulation type × Stimulation polarity	0.04	0.01	1.00	10,285.40	19,267.43	∞*	[0.03, 0.06]
Group × Stimulation type × Stimulation polarity	-0.05	0.01	1.00	15,454.88	23,335.56	∞*	[-0.07, -0.03]
SDMT n correct × Group × Stimulation type × Stimulation polarity	0.06	0.01	1.00	12,180.34	20,716.77	∞*	[0.04, 0.08]

Note. Group-level subject intercepts Estimate: 0.14 (95%CI [0.11, 0.17]). Rhat: 1, Bulk ESS: 5227.36; Tail ESS: 9921.92. Rhat values should be close to 1 to indicate convergence. Bulk ESS and Tail ESS indicate the effective sample size of the MCMC chains. Evidence ratio indicate the ratio of draws that were in the direction of the estimate relative to the number of draws that were in the opposite direction, e.g., for the TMT-A time effect, there were 155.86 times more positive draws than negative draws. Simulations in linear models show, that an evidence ratio of 19 is equivalent to p-value of 0.05 (Makowski et al.).[1] SE=Standard error. ESS=Effective sample size. TMT-A=Trail making test version A. SDMT=Symbol-digits-modalities-test.

* Evidence ratios equal to ∞ indicate that all posterior draws were in favor of the estimate direction.

Supplementary Table 8

Population-level effects of the exploratory response latency model with SDMT z-scores from Smith et al.[2]

Parameter	*Estimate*	*SE*	*Rhat*	*Bulk ESS*	*Tail ESS*	*Evidence ratio*	*95% CI*
Intercept	0.56	0.04	1.00	3,884.33	7,416.25	∞*	[0.47, 0.64]
Session	-0.12	0.00	1.00	51,161.18	25,510.37	∞*	[-0.13, -0.12]
TMT-A time	0.09	0.03	1.00	4,464.91	7,974.35	443.44	[0.03, 0.15]
SDMT z-score	-0.06	0.06	1.00	3,823.58	7,954.16	4.51	[-0.18, 0.06]
Group	0.09	0.08	1.00	3,547.80	7,038.11	6.80	[-0.07, 0.26]
Stimulation type	-0.03	0.00	1.00	22,592.72	26,760.26	∞*	[-0.04, -0.02]
Stimulation polarity	-0.12	0.06	1.00	3,004.09	6,136.42	32.00	[-0.24, 0.01]
SDMT z-score × Group	0.01	0.08	1.00	3,639.20	7,722.49	1.22	[-0.14, 0.16]
SDMT z-score × Stimulation type	-0.03	0.01	1.00	14,099.24	21,756.18	∞*	[-0.04, -0.02]
Group × Stimulation type	0.02	0.01	1.00	15,344.33	22,139.87	50.81	[0.00, 0.03]
SDMT z-score × Stimulation polarity	-0.05	0.07	1.00	3,911.77	8,200.19	3.17	[-0.19, 0.09]
Group × Stimulation polarity	-0.04	0.11	1.00	3,379.99	6,013.64	1.84	[-0.26, 0.18]
Stimulation type × Stimulation polarity	0.07	0.01	1.00	22,533.99	25,630.98	∞*	[0.06, 0.08]
SDMT z-score × Group × Stimulation type	0.01	0.01	1.00	11,663.75	18,732.62	8.48	[-0.01, 0.02]
SDMT z-score × Group × Stimulation polarity	-0.06	0.09	1.00	4,080.79	8,110.54	2.62	[-0.24, 0.13]

Supplementary Table 8

Population-level effects of the exploratory response latency model with SDMT z-scores from Smith et al.[2]

Parameter	*Estimate*	*SE*	*Rhat*	*Bulk ESS*	*Tail ESS*	*Evidence ratio*	*95% CI*
SDMT z-score × Stimulation type × Stimulation polarity	0.04	0.01	1.00	14,179.99	20,977.33	∞*	[0.03, 0.06]
Group × Stimulation type × Stimulation polarity	0.00	0.01	1.00	15,181.18	20,210.41	1.54	[-0.02, 0.02]
SDMT z-score × Group × Stimulation type × Stimulation polarity	0.06	0.01	1.00	11,865.37	18,779.86	∞*	[0.04, 0.08]

Note. Group-level subject intercepts Estimate: 0.17 (95%CI [0.14, 0.21]). Rhat: 1, Bulk ESS: 5117.68; Tail ESS: 10861.7. Rhat values should be close to 1 to indicate convergence. Bulk ESS and Tail ESS indicate the effective sample size of the MCMC chains. Evidence ratio indicate the ratio of draws that were in the direction of the estimate relative to the number of draws that were in the opposite direction, e.g., for the TMT-A time effect, there were 96.17 times more positive draws than negative draws. Simulations in linear models show, that an evidence ratio of 19 is equivalent to p-value of 0.05 (Makowski et al.).[1]

SE=Standard error. ESS=Effective sample size. TMT-A=Trail making test version A. SDMT=Symbol-digits-modalities-test.

* Evidence ratios equal to ∞ indicate that all posterior draws were in favor of the estimate direction.

Supplementary Table 9

Population-level effects of the response latency model in pwRMS.

Parameter	Estimate	*SE*	Rhat	Bulk ESS	Tail ESS	Evidence ratio	95% CI
Intercept	0.64	0.05	1.00	8,897.20	11,061.25	∞*	[0.54, 0.73]
Stimulation type	0.00	0.01	1.00	22,009.93	16,863.68	1.76	[-0.01, 0.01]
Stimulation polarity	-0.04	0.06	1.00	9,168.97	11,106.05	2.75	[-0.16, 0.08]
SDMT n correct	-0.12	0.05	1.00	11,243.72	12,201.57	157.94	[-0.21, -0.03]
TMT-A time	0.07	0.03	1.00	12,411.58	12,053.85	116.07	[0.01, 0.13]
Session	-0.13	0.00	1.00	23,680.12	15,989.75	∞*	[-0.13, -0.12]
Stimulation type × Stimulation polarity	-0.01	0.01	1.00	22,014.91	18,107.13	7.87	[-0.02, 0.01]
Stimulation type × SDMT n correct	-0.02	0.01	1.00	22,129.22	18,084.50	799.00	[-0.03, -0.01]
Stimulation polarity × SDMT n correct	-0.09	0.05	1.00	11,493.03	12,605.57	19.05	[-0.20, 0.02]
Stimulation type × Stimulation polarity × SDMT n correct	0.10	0.01	1.00	21,703.93	17,318.63	∞*	[0.09, 0.12]

Note. Subject group-level intercepts Estimate: 0.13 (95%CI [0.1, 0.18]). Rhat: 1, Bulk ESS: 7694.48; Tail ESS: 11714.05. Rhat values should be close to 1 to indicate convergence. Bulk ESS and Tail ESS indicate the effective sample size of the MCMC chains. Evidence ratio indicate the ratio of draws that were in the direction of the estimate relative to the number of draws that were in the opposite direction, e.g., for the TMT-A time effect, there were 116.07 times more positive draws than negative draws. Simulations in linear models show, that an evidence ratio of 19 is equivalent to p-value of 0.05 (Makowski et al.).[1] SE=Standard error. ESS=Effective sample size. TMT-A=Trail making test version A. SDMT=Symbol-digits-modalities-test. pwRMS=patients with relapsing multiple scerlosis.

* Evidence ratios equal to ∞ indicate that all posterior draws were in favor of the estimate direction.

Supplementary Table 10

Population-level effects of the response latency model in the subgroup of pwRMS.

Parameter	*Estimate*	*SE*	*Rhat*	*Bulk ESS*	*Tail ESS*	*Evidence ratio*	*95% CI*
Intercept	0.65	0.07	1.00	11,825.90	12,732.16	∞*	[0.51, 0.78]
Stimulation type	-0.01	0.01	1.00	20,071.69	17,042.01	21.20	[-0.03, 0.00]
Stimulation polarity	-0.16	0.09	1.00	11,980.47	13,384.06	19.82	[-0.34, 0.03]
SDMT n correct	-0.04	0.05	1.00	12,679.41	12,062.49	4.35	[-0.14, 0.06]
TMT-A time	0.09	0.04	1.00	13,850.91	13,417.16	120.21	[0.02, 0.16]
Session	-0.12	0.00	1.00	25,460.13	15,710.52	∞*	[-0.13, -0.11]
Stimulation type × Stimulation polarity	0.07	0.01	1.00	20,472.64	16,321.97	∞*	[0.05, 0.09]
Stimulation type × SDMT n correct	-0.02	0.01	1.00	20,499.74	16,872.50	23,999.00	[-0.03, -0.01]
Stimulation polarity × SDMT n correct	-0.11	0.06	1.00	13,473.99	12,876.92	28.63	[-0.22, 0.01]
Stimulation type × Stimulation polarity × SDMT n correct	0.10	0.01	1.00	20,340.12	17,566.10	∞*	[0.09, 0.12]

Note. Subject group-level intercepts Estimate: 0.17 (95%CI [0.13, 0.23]). Rhat: 1, Bulk ESS: 6793.97; Tail ESS: 11128.06. Rhat values should be close to 1 to indicate convergence. Bulk ESS and Tail ESS indicate the effective sample size of the MCMC chains. Evidence ratio indicate the ratio of draws that were in the direction of the estimate relative to the number of draws that were in the opposite direction, e.g., for the TMT-A time effect, there were 120.21 times more positive draws than negative draws. Simulations in linear models show, that an evidence ratio of 19 is equivalent to p-value of 0.05 (Makowski et al.).[1] SE=Standard error. ESS=Effective sample size. TMT-A=Trail making test version A. SDMT=Symbol-digits-modalities-test. pwRMS=patients with relapsing multiple sclerosis.

* Evidence ratios equal to ∞ indicate that all posterior draws were in favor of the estimate direction.

Supplementary Table 11

Population-level effects of the cumulative probit model for the intensity of adverse effects.

Parameter	*Estimate*	*SE*	*Rhat*	*Bulk ESS*	*Tail ESS*	*Evidence ratio*	*95% CI*
$\text{Intercept}_{\text{none, mild}}$	1.03	0.29	1.00	2,276.56	1,915.80	999.00	[0.44, 1.62]
$\text{Intercept}_{\text{mild, moderate}}$	1.95	0.30	1.00	2,349.60	2,001.08	∞^{*}	[1.32, 2.55]
$\text{Intercept}_{\text{moderate, strong}}$	2.85	0.34	1.00	2,726.72	1,989.82	∞^{*}	[2.18, 3.56]
Subject group	-0.14	0.19	1.00	3,202.38	3,062.38	3.40	[-0.50, 0.23]
Stimulation type	-0.01	0.18	1.00	2,861.39	2,863.89	1.09	[-0.37, 0.36]
Active stimulation	0.03	0.10	1.00	8,556.63	3,000.71	1.64	[-0.18, 0.23]
Itching	0.21	0.39	1.00	2,607.32	2,095.62	3.24	[-0.64, 0.99]
Burning	0.30	0.37	1.00	3,161.52	2,491.45	4.87	[-0.48, 1.05]
Pain	-0.11	0.38	1.00	2,880.37	1,847.70	1.80	[-0.89, 0.72]
Metallic taste	-1.66	0.59	1.00	3,926.62	2,333.91	132.33	[-2.82, -0.42]
Fatigue	0.37	0.39	1.00	2,493.29	2,062.28	7.05	[-0.51, 1.12]
Other	0.37	0.38	1.00	2,217.43	1,981.60	6.68	[-0.47, 1.12]
Subject group × Stimulation type	0.11	0.26	1.00	2,743.54	3,012.50	2.01	[-0.40, 0.62]

Note. Group level intercepts $SD_{subject}$=0.34, 95%CI=[0.14, 0.54]; $SD_{sensation}$=0.27, 95%CI=[0.01, 0.93]. Rhat values should be close to 1 to indicate convergence. Bulk ESS and Tail ESS indicate the effective sample size of the MCMC chains. Evidence ratio indicate the ratio of draws that were in the direction of the estimate relative to the number of draws that were in the opposite direction, e.g., for the TMT-A time effect, there were 239 times more positive draws than negative draws. Simulations in linear models show, that an evidence ratio of 19 is equivalent to a p-value of 0.05 (Makowski et al.).[1]

* Evidence ratios equal to ∞ indicate that all posterior draws were in favor of the estimate direction.

Supplementary Table 12

Means and standard deviation of the PANAS sum scores for the experimental groups.

Valence	Time point	*Control*								*pwRMS*							
		anodal				*cathodal*				*anodal*				*cathodal*			
		Sham		*Active*		*Sham*		*Active*		*Sham*		*Active*		*Sham*		*Active*	
		M	*SD*	*M*	*SD*	*M*	*SD*	*M*	*SD*	*M*	*SD*	*M*	*SD*	*M*	*SD*	*M*	*SD*
positive	pre	22.00	6.00	23.38	7.54	24.31	6.88	23.00	8.56	23.29	7.01	23.44	6.26	22.64	6.85	22.64	7.27
	post	22.38	7.93	22.94	5.79	21.50	9.36	21.00	8.64	20.79	4.59	19.75	5.71	19.50	8.85	19.50	7.06
negative	pre	1.81	2.79	1.75	4.64	0.44	0.51	0.62	0.81	1.50	1.95	1.69	2.02	2.86	3.57	2.36	2.24
	post	0.94	1.61	0.94	3.75	0.00	0.00	0.00	0.00	1.36	1.82	0.69	0.70	1.14	1.41	1.79	2.78

Note. M=mean.SD=standard deviation. pwRMS=patients with relapsing multiple sclerosis.

Supplementary Table 13

Fixed effects of the hurdle Gaussian PANAS model.

Parameter	*Estimate*	*SE*	*Rhat*	*Bulk ESS*	*Tail ESS*	*Evidence ratio*	*95% CI*
Intercept	20.57	0.89	1.00	8,284.64	9,205.50	∞^*	[18.81, 22.31]
$\text{Intercept}_{\text{hurdle}}$	-7.53	2.19	1.00	9,271.07	5,031.63	∞^*	[-13.02, -4.63]
Valence	-11.36	0.75	1.00	13,662.44	9,271.35	∞^*	[-12.81, -9.87]
Time point	0.26	0.65	1.00	18,761.53	9,383.37	1.88	[-1.00, 1.54]
Group	0.09	0.79	1.00	11,748.41	10,060.58	1.19	[-1.45, 1.63]
Stimulation polarity	0.41	0.79	1.00	11,325.54	9,477.47	2.29	[-1.15, 1.96]
Stimulation type	0.19	0.53	1.00	19,845.19	9,228.64	1.76	[-0.86, 1.25]
Valence × Time point	-1.40	0.81	1.00	22,119.99	8,593.14	22.26	[-2.99, 0.20]
Valence × Group	-3.22	0.78	1.00	20,402.31	8,781.25	∞^*	[-4.73, -1.68]
Time point × Group	-1.06	0.73	1.00	17,997.89	9,377.17	12.44	[-2.49, 0.38]
Valence × Stimulation polarity	-2.79	0.79	1.00	19,649.19	8,789.27	2,999.00	[-4.34, -1.26]
Time point × Stimulation polarity	-0.41	0.74	1.00	19,868.60	9,220.28	2.47	[-1.84, 1.02]
Group × Stimulation polarity	0.17	0.86	1.00	13,810.08	9,591.41	1.37	[-1.48, 1.87]
Time point × Stimulation type	0.21	0.68	1.00	19,254.29	9,412.61	1.66	[-1.11, 1.55]
Valence × Time point × Group	-0.39	0.88	1.00	22,328.46	8,989.21	2.03	[-2.11, 1.31]

Supplementary Table 13

Fixed effects of the hurdle Gaussian PANAS model.

Parameter	*Estimate*	*SE*	*Rhat*	*Bulk ESS*	*Tail ESS*	*Evidence ratio*	*95% CI*
Valence × Time point × Stimulation polarity	0.36	0.91	1.00	21,523.19	9,358.34	1.90	[-1.43, 2.15]
Valence × Group × Stimulation polarity	-0.26	0.85	1.00	20,364.00	9,241.01	1.65	[-1.94, 1.38]
Time point × Group × Stimulation polarity	-0.33	0.82	1.00	20,932.56	9,891.50	1.92	[-1.95, 1.28]
Valence × Time point × Group × Stimulation polarity	0.35	0.93	1.00	22,480.87	9,046.95	1.78	[-1.47, 2.16]
$\text{Valence}_{\text{hurdle}}$	7.67	2.19	1.00	9,223.14	5,009.22	∞*	[4.75, 13.15]

Note. Subject group-level intercepts Estimate: 4.04 (95%CI [3.21, 4.99]). Rhat: 1, Bulk ESS: 4858.01; Tail ESS7061.43. Rhat values should be close to 1 to indicate convergence. Buld ESS and Tail ESS indicate the effective sample size of the MCMC chains. Evidence ratio indicate the ratio of draws that were in the direction of the estimate relative to the number of draws that were in the opposite direction, e.g., for the Time point effect, there were 1.88 times more positive draws than negative draws. Simulations in linear models show, that an evidence ratio of 19 is equivalent to a p-value of 0.05 (Makowski et al.).[1]

SE=Standard error. ESS=Effective sample size.

* Evidence ratios equal to ∞ indicate that all posterior draws were in favor of the estimate direction.

Supplementary Table 14

Fixed effects of the categorical model predicting blinding guess.

Parameter	Estimate	*SE*	Rhat	Bulk ESS	Tail ESS	Evidence ratio	95% CI
Intercept$_{t1}$	0.02	0.60	1.00	13,635.81	9,841.71	1.06	[-1.15, 1.18]
Intercept$_{t2}$	0.53	0.54	1.00	12,311.48	10,292.63	5.10	[-0.54, 1.63]
Subject group$_{t1}$	-0.53	0.67	1.00	12,929.49	9,602.22	3.64	[-1.85, 0.77]
Stimulation type$_{t1}$	-0.30	0.66	1.00	12,197.62	9,508.79	2.08	[-1.61, 0.98]
Active stimulation$_{t1}$	-0.57	0.66	1.00	12,833.65	9,288.15	4.12	[-1.87, 0.72]
Subject group × stimulation type$_{t1}$	-0.69	0.79	1.00	13,342.22	10,197.73	4.33	[-2.25, 0.84]
Subject group × active stimulation$_{t1}$	-0.69	0.79	1.00	13,809.97	9,314.24	4.15	[-2.27, 0.85]
Stimulation type × active stimulation$_{t1}$	0.91	0.75	1.00	12,100.20	10,195.56	7.83	[-0.54, 2.40]
Subject group × stimulation type × active stimulation$_{t1}$	0.08	0.86	1.00	14,393.74	9,470.31	1.18	[-1.63, 1.76]
Subject group$_{t2}$	-0.91	0.62	1.00	11,975.77	9,390.54	13.32	[-2.12, 0.28]
Stimulation type$_{t2}$	0.36	0.61	1.00	12,293.27	9,451.60	2.61	[-0.84, 1.56]
Active stimulation$_{t2}$	-0.43	0.61	1.00	12,548.31	9,424.39	3.11	[-1.65, 0.79]
Subject group × stimulation type$_{t2}$	-0.42	0.74	1.00	12,905.71	9,800.49	2.52	[-1.86, 1.02]
Subject group × active stimulation$_{t2}$	0.53	0.71	1.00	11,864.54	8,666.52	3.43	[-0.87, 1.95]
Stimulation type × active stimulation$_{t2}$	-1.09	0.74	1.00	12,827.28	9,196.86	13.00	[-2.52, 0.36]

Supplementary Table 14

Fixed effects of the categorical model predicting blinding guess.

Parameter	Estimate	*SE*	Rhat	Bulk ESS	Tail ESS	Evidence ratio	95% CI
Subject group × stimulation type × active $stimulation_{t2}$	0.65	0.82	1.00	12,209.06	9,092.66	3.77	[-0.97, 2.25]

Note. Rhat values should be close to 1 to indicate convergence. Bulk ESS and Tail ESS indicate the effective sample size of the MCMC chains. Evidence ratio indicate the ratio of draws that were in the direction of the estimate relative to the number of draws that were in the opposite direction, e.g., for the TMT-A time effect, there were 239 times more positive draws than negative draws. Simulations in linear models show, that an evidence ratio of 19 is equivalent to a p-value of 0.05 (Makowski et al.).[1] Note that the model fits an every parameter twice: for differences between "do not know"- and "t1"-responses and for differences between "do not know"- and "t2"-responses.

Supplementary References

1. Makowski D, Ben-Shachar MS, Chen SHA, Lüdecke D. Indices of Effect Existence and Significance in the Bayesian Framework. *Front Psychol*. 2019;10. Accessed February 7, 2023. https://www.frontiersin.org/articles/10.3389/fpsyg.2019.02767

2. Smith A. Symbol-Digit-Modalities Test (SDMT) manual (revised) Western psychological services. *Los Angel*. Published online 1982:10.
